# Microbial cell factories for gamma-linolenic acid biosynthesis: metabolic engineering, downstream processing, and future perspectives

**DOI:** 10.1186/s40643-026-01146-w

**Published:** 2026-10-17

**Authors:** Jie Qiao, Wei Wang, Peiyao Duan, Zhili Zhang, Rongrong Yang, Haiyang Cui, Xiujuan Li

**Affiliations:** 1 State Key Laboratory of Microbial Technology, School of Food Science and Pharmaceutical Engineering, Nanjing Normal University, Nanjing, 210023 China; 2 School of Life Sciences, Nanjing Normal University, Nanjing, 210097 China

**Keywords:** Gamma-linolenic acid (GLA), Microbial lipids, Metabolic engineering, Biosynthesis, Machine learning

## Abstract

Gamma-linolenic acid (GLA) is a vital anti-inflammatory polyunsaturated fatty acid, yet its traditional supply from plant seeds such as evening primrose and borage is constrained by low yields and climatic instability. Consequently, microbial cell factories present a sustainable and season-independent alternative for scalable GLA production. This review critically synthesizes recent biotechnological advancements in utilizing oleaginous microorganisms as production platforms. It systematically elucidates the microbial lipid biosynthesis pathways and evaluates strategies to enhance GLA accumulation, with particular emphasis on rational metabolic engineering. Furthermore, emerging technologies for microbial lipid production, alongside the latest progress in industrial-scale applications, are thoroughly examined. Finally, comprehensive insights are provided to guide the optimization of the entire value chain, ranging from upstream strain engineering to downstream processing.

**Graphical abstract:**

**Figure:**
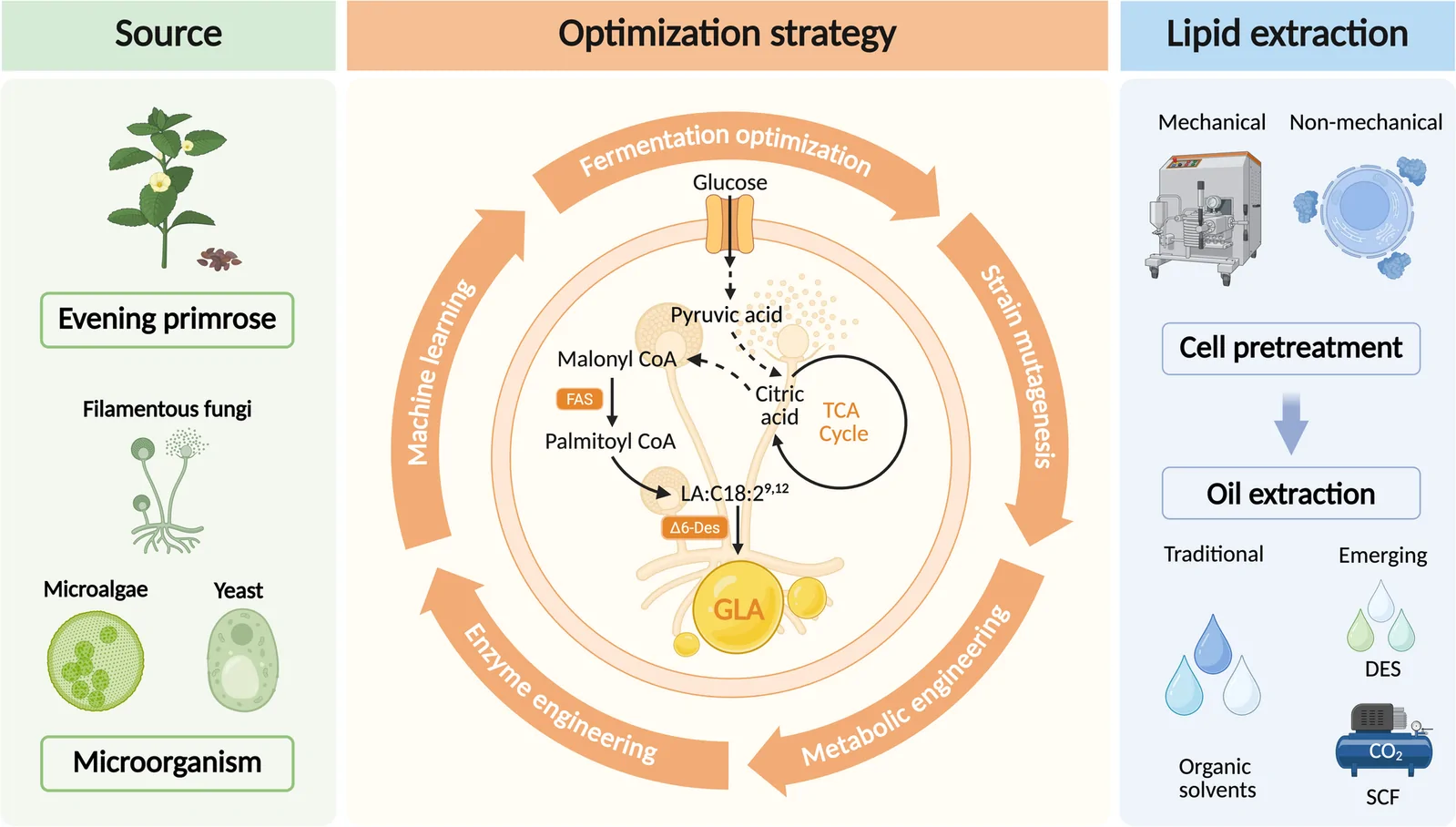

## Introduction

Polyunsaturated fatty acids (PUFAs) are important components of functional lipids and play diverse roles in membrane organization, cellular signaling, and the generation of bioactive lipid mediators (Markovic et al. 2020, Xie et al. 2023, Liu et al. 2023). Among them, ω-3 and ω-6 PUFAs have attracted particular attention because of their close association with human nutrition and health (Liu et al. 2023, Roychoudhury 2020). Gamma-linolenic acid (GLA; all-cis-6,9,12-octadecatrienoic acid, C18:3, Fig. 1) is an important member of the ω-6 PUFA family. GLA is biosynthesized from linoleic acid (LA) through Δ6-desaturation and can subsequently be elongated to dihomo-γ-linolenic acid (DGLA), a precursor of several bioactive lipid mediators. GLA is incorporated into membrane phospholipids, thereby modulating cellular signaling pathways and membrane biophysical properties (Dyall et al. 2022). These metabolic and physiological functions, together with its applications in nutraceutical, healthcare, and cosmetic products, have stimulated increasing interest in the sustainable production of GLA.

**Fig. 1:**
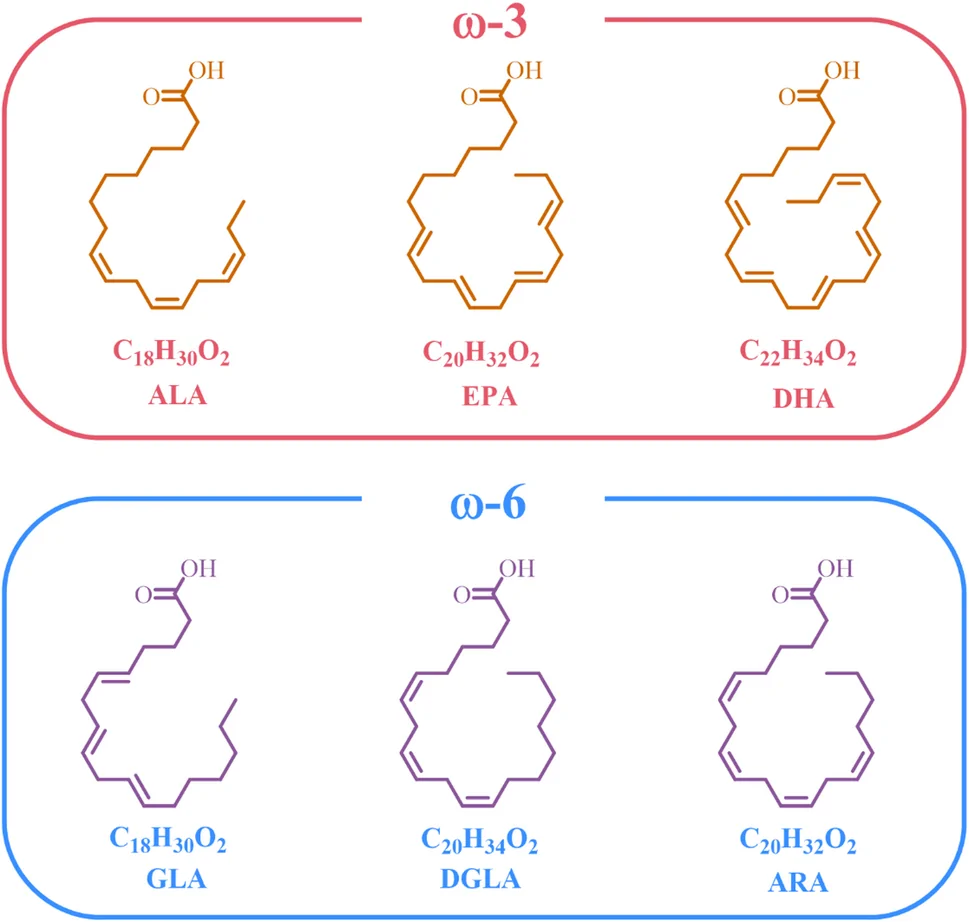
Molecular structure of ω-3 and ω-6 fatty acids. ALA, α-linolenic acid; EPA, eicosapentaenoic acid; DHA, docosahexaenoic acid; GLA, γ-linolenic acid; DGLA, dihomo-γ-linolenic acid; ARA, arachidonic acid

Commercial GLA is currently derived primarily from plant seed oils, particularly evening primrose, borage, and blackcurrant oils. However, the GLA content derived from plant sources is relatively low, with even the highest-quality borage oil containing only 17–25% GLA (Latifi et al. 2025). In addition, plant-based production is constrained by long cultivation cycles and susceptibility to seasonal and climatic variations, which limit production stability and scalability. Although chemical synthesis can circumvent some of these agricultural limitations, its high cost, operational complexity, and limited scalability restrict broader application. These constraints have stimulated growing interest in microbial production systems as controllable and season-independent alternatives for GLA production. Oleaginous filamentous fungi, including *Mucor*, *Mortierella*, and *Cunninghamella*, as well as selected microalgae and engineered yeasts, have therefore emerged as promising microbial platforms for GLA biosynthesis (Kennedy et al. 1993, Chen and Chang 1996, Shimiziu et al. 1988).

Advances in genetic, metabolic, and enzyme engineering have provided new opportunities to further improve microbial GLA production by enabling targeted modification of lipid biosynthetic pathways (Fazili et al. 2022, Wang et al. 2023). In addition to engineering native oleaginous producers, heterologous reconstruction of GLA biosynthesis in chassis cells has further expanded the available production platforms (Sun et al. 2017). Nevertheless, the industrial competitiveness of microbial GLA remains constrained by production capacity and overall process costs (Szczepańska et al. 2022). Current efforts therefore extend beyond strain improvement to the use of renewable and low-cost feedstocks (Mhlongo et al. 2021), more efficient and environmentally sustainable lipid extraction technologies (William et al. 2020, Araujo et al. 2021, Rivera-Baez et al. 2026), and data-driven approaches for fermentation and process optimization (Duong-Trung et al. 2023). In this review, we summarize the biological functions and major sources of GLA, describe its biosynthetic pathways in oleaginous microorganisms, and discuss recent advances in fermentation optimization, strain improvement, metabolic and enzyme engineering, machine learning (ML), and downstream processing. Finally, the major challenges and future perspectives for the industrial production of microbial GLA are discussed.

## Biological functions of GLA

**Fig. 2:**
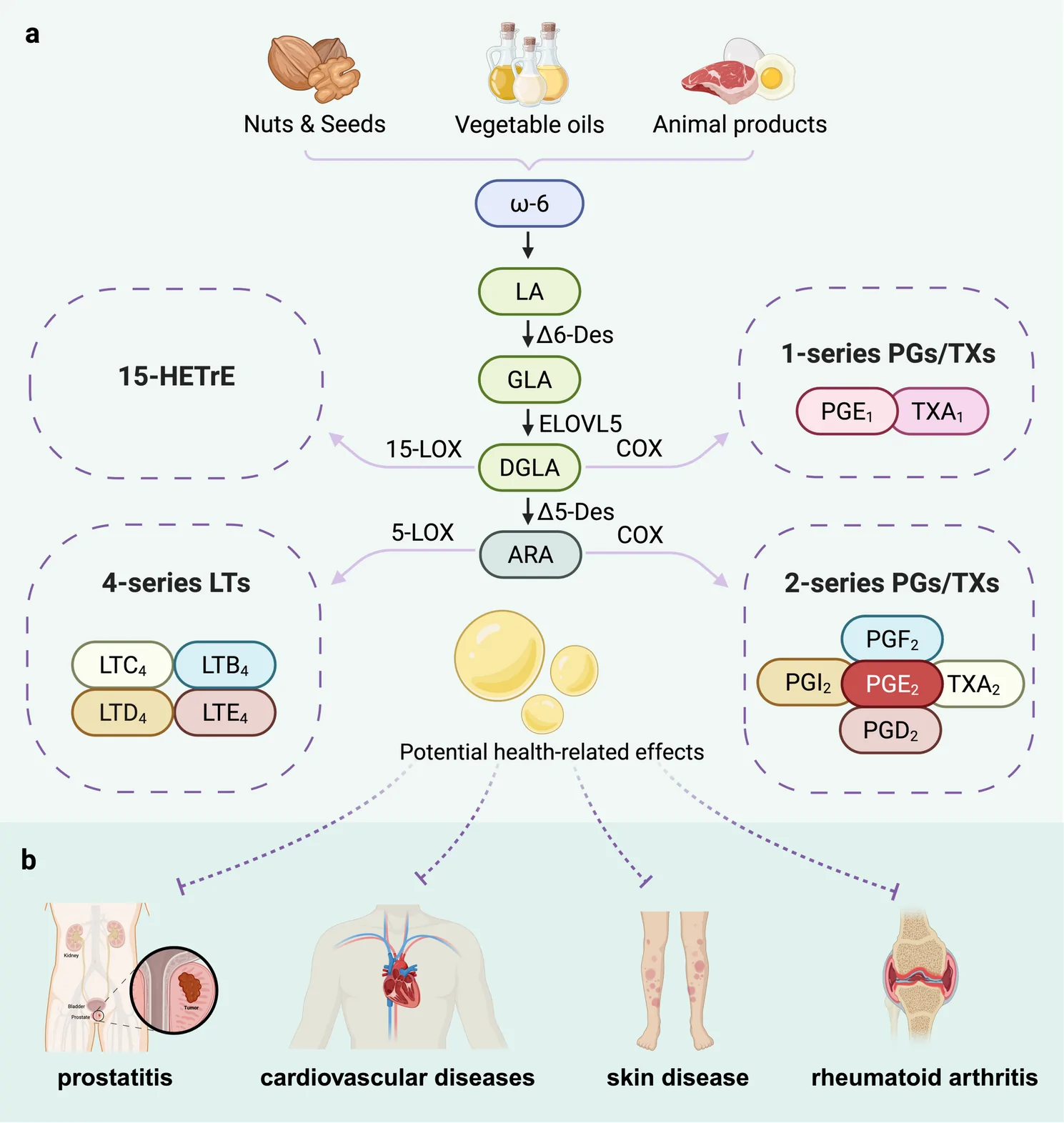
Overview of GLA metabolism and its potential health-related effects. **a** Major metabolic pathways leading from LA to GLA, DGLA, and ARA and the formation of downstream eicosanoids through desaturation, elongation, COX, and LOX pathways. **b** Representative health-related effects associated with GLA and its downstream metabolites. LA, linoleic acid; GLA, γ-linolenic acid; DGLA, dihomo-γ-linolenic acid; ARA, arachidonic acid; ELOVL5, elongation of very long-chain fatty acids protein 5; Δ6-Des, Δ6-desaturase; Δ5-Des, Δ5-desaturase; COX, cyclooxygenase; 5-LOX, 5-lipoxygenase; 15-LOX, 15-lipoxygenase; 15-HETrE, 15-hydroxyeicosatrienoic acid; PGs, prostaglandins; TXs, thromboxanes; LTs, leukotrienes; PGE, prostaglandin E; PGF, prostaglandin F; PGD, prostaglandin D; PGI, prostacyclin; TXA, thromboxane A

Physiologically, GLA serves as a critical checkpoint in polyunsaturated fatty acid metabolism (Fig. 2). Catalyzed by elongation of very long-chain fatty acids protein 5 (ELOVL5), GLA is rapidly elongated to dihomo-γ-linolenic acid (DGLA, all-cis-8,11,14, C20:3). DGLA functions as a central precursor with divergent metabolic fates: it can be further desaturated to arachidonic acid (ARA, all-cis-5,8,11,14, C20:4) via Δ5-desaturase, or directly metabolized by cyclooxygenases (COX) and lipoxygenases (LOX) into bioactive lipid mediators.

The therapeutic efficacy of GLA largely stems from its ability to shift the metabolic balance towards DGLA-derived eicosanoids. Specifically, DGLA is converted into series-1 prostaglandins, such as prostaglandin E_1_ (PGE_1_), and thromboxane A_1_(TXA_1_) as well as 15-hydroxyeicosatrienoic acid (15-HETrE). Unlike the generally pro-inflammatory series-2 eicosanoids (PGE_2_, TXA_2_) derived from ARA, PGE_1_ exerts potent anti-inflammatory and vasodilatory effects (Mustonen and Nieminen 2023), while modulating platelet aggregation and thrombosis risks (Szczuko et al. 2021).

Furthermore, GLA and its metabolites exhibit significant anticarcinogenic properties. Studies indicate that GLA modulation of COX and LOX pathways influences cell proliferation and differentiation (Vishnupriya et al. 2021), effectively suppressing tumor progression in breast and colorectal cancers (Jackson et al. 2023). For instance, the metabolite 15-HETrE is known to inhibit cancer cell proliferation and metastasis. Conversely, insufficient endogenous levels of GLA and its downstream metabolites have been associated with the pathogenesis of inflammatory and cardiovascular disorders. Consequently, GLA has attracted considerable attention as a bioactive dietary fatty acid with potential therapeutic and health-promoting effects across a range of inflammatory and metabolic disorders (Cui et al. 2018, Sharifi et al. 2024).

### Cardioprotective effects of GLA

In Western countries, circulatory system diseases, particularly ischemic heart disease (IHD), remain the leading cause of morbidity and mortality, surpassing the combined burden of infectious, maternal, and nutritional disorders (Zaman et al. 2025). Beyond systemic inflammation and oxidative stress, specific dysregulation in fatty acid metabolism has emerged as a critical driver of atherosclerosis and cardiovascular disease (CVD) pathogenesis (Yang et al. 2025). Epidemiological evidence supports this link. For instance, Yang et al. established a significant inverse correlation between total serum ω-6 fatty acid concentrations and CVD risk. Corroborating these findings, higher dietary intake of ω-6 PUFAs has been associated with a reduced incidence of myocardial infarction (Xu et al. 2025). While current evidence does not validate GLA as a definitive curative agent, studies confirm that supplementation favorably modulates key cardiovascular biomarkers (Shramko et al. 2020).

In spontaneously hypertensive models, St. Louis et al. demonstrated that GLA administration could reversibly normalize blood pressure, with hypertension recurring upon treatment cessation (St Louis et al. 1992). Mechanistically, this hypotensive effect is linked to the renal conversion of GLA into prostaglandin E_2_ (PGE_2_), which modulates renal hemodynamics and interacts with vasoactive mediators (Zhou et al. 2021). Beyond hemodynamic regulation, GLA exhibits potent hypolipidemic activity. Dietary supplementation has been shown to significantly reduce serum triacylglycerol and cholesterol concentrations in hyperlipidemic mice (Ide and Origuchi 2019). Regarding hemostasis, Guivernau reported that the GLA metabolite DGLA inhibits thromboxane A_2_ (TXA_2_) synthase, thereby maintaining normal platelet function. Furthermore, DGLA exerts antithrombotic effects through the generation of bioactive lipid mediators, including PGE_1_ and 12(S)-HETrE, which can suppress platelet activation and aggregation and thereby limit thrombus formation (Stanger and Holinstat 2023). At the cellular level, Fan et al. observed that GLA elevates DGLA accumulation in peritoneal macrophages and stimulates PGE_1_ secretion. This upregulation of PGE_1_ exerts critical anti-atherosclerotic effects by inhibiting vascular smooth muscle cell proliferation (Sergeant et al. 2016), suppressing superoxide anion generation, and activating the fibrinolytic system (Ide and Origuchi 2019).

### Antidiabetic effects of GLA

As an essential component of the cell membrane’s phospholipid bilayer, GLA can enhance insulin receptor sensitivity and promote insulin secretion by increasing adenylyl cyclase activity via its downstream metabolites, prostaglandins (Mititelu et al. 2024, Yuan et al. 2026). Clinical observations indicate that GLA supplementation optimizes prostaglandin profiles in diabetic patients, thereby facilitating improved glycemic control (Xu et al. 2025). Crucially, diabetes is frequently characterized by compromised Δ5/6-desaturase activity (Jia et al. 2022). This enzymatic bottleneck restricts the endogenous biosynthesis of GLA, DGLA, and ARA, a deficiency directly implicated in the pathogenesis of diabetic complications (Yang et al. 2024). Consequently, bypassing this metabolic block via exogenous GLA intake serves as a vital therapeutic strategy. In rat models, Julu demonstrated that GLA intake significantly ameliorated nerve conduction velocity deficits (Julu 1988), a finding corroborated by Cameron (Cameron and Cotter 1996), who reported an approximate 20% reduction in deficits at optimal dosages. Notably, co-administration of GLA with antioxidants exhibits synergistic efficacy in mitigating these complications.

### Anti-cancer and anti-inflammatory effects of GLA

GLA regulates cell apoptosis by increasing the production of reactive oxygen species and lipid peroxidation products, while simultaneously decreasing the activity of mitochondrial respiratory chain complexes I, III and IV, as well as the mitochondrial membrane potential, to induce the death of cancer cells (Montecillo-Aguado et al. 2023). This anticancer efficacy is substantiated by extensive in vivo and in vitro studies (Kapoor and Nair 2020). Crucially, GLA has demonstrated selective antitumor activity across several cancer models, including hepatocellular carcinoma, breast cancer, and glioblastoma, while showing relatively limited cytotoxicity toward normal cells under experimental conditions (Zikic et al. 2025). Beyond its oncological applications, GLA also possesses significant broad-spectrum antimicrobial and anti-inflammatory capabilities.

The anti-inflammatory properties of GLA were initially discovered through observations of the beneficial effects of long-term evening primrose oil supplementation in mitigating rheumatoid arthritis symptoms (Sharifi et al. 2024). Subsequently, GLA has gained recognition as a therapeutic supplement for rheumatoid arthritis (Das 2023). Mechanistically, this therapeutic effect is driven by the metabolic accumulation of DGLA, which acts as a competitive substrate for COX and LOX enzymes against ARA. This competition shifts the eicosanoid profile: it promotes the synthesis of anti-inflammatory prostaglandins (e.g., PGE_1_) while the DGLA derivative, 15-HETrE, actively inhibits the biosynthesis of the potent pro-inflammatory mediator leukotriene B_4_ (LTB_4_) (Sharifi et al. 2024). Leveraging these metabolic effects, GLA-containing formulations have been investigated as potential adjunctive interventions for a range of inflammatory conditions, including acute respiratory distress syndrome (ARDS), atopic dermatitis, rheumatoid arthritis, and other inflammatory disorders. In ARDS, enteral nutrition containing eicosapentaenoic acid (EPA) and GLA was associated with reduced pulmonary inflammation, improved oxygenation, and shorter durations of mechanical ventilation and intensive care unit stay (Sharifi et al. 2024, Shirai et al. 2015, Weber et al. 2023).

## Sources of GLA

### Plant sources of GLA

While constrained by the limitations discussed earlier, plant seed oils remain the dominant commercial source of GLA (Afzal et al. 2022). Currently, the major commercial sources of GLA include the seed oils from four plants: evening primrose (*Oenothera biennis* L.), borage (*Borago officinalis* L.), blackcurrants (*Ribes nigrum* L.), and hemp (*Cannabis sativa* L.) (Golovenko et al. 2021) . Although *O. biennis* is the traditional standard (18–25% total lipid; 7–10% GLA*)* (Serini et al. 2009), *B. officinalis* possesses superior commercial traits, exhibiting the highest natural GLA fraction (17–25%) and a shorter cultivation cycle (Guil-Guerrero et al. 2018). Other sources, such as *R. nigrum* (3–23% GLA) and minor taxa like *Pinus armandii*, offer alternative reservoirs. Detailed compositional profiles of representative GLA-rich plants are summarized in Table 1.

**Table 1:** Major plant species high in GLA

Family	Genus and species	Total oil content of seeds (%)	GLA content of oil (%)	References
Onagraceae	*Oenothera biennis* L.	20–30	7–10	Mercuri et al. (1966), Wu et al. (2017)
	*Oenothera grandiflora*	4	9.3	Kapoor and Nair (2020)
Boraginaceae	*Anchusa azurea* Mill.	21	8–13	Gazem and Chandrashekariah (2014), Guil‐Guerrero et al. (2006), Miller et al. (1968)
	*Anchusa capellii*	24.2	12	Guil-Guerrero et al. (2017)
	*Anchusa undulata* L.	16.9–29.4	10.3–12.3	Jia et al. (2022), Barabino et al. (2003)
	*Anchusa crispa*	16.9–18.6	11.7–12.3	Guil-Guerrero et al. (2017)
	*Borago officinalis*	28–38	17–25	Gazem et al. (2014), Guil-Guerrero et al. (2001, 2017)
	*Borago pygmaea*	21.9	22.9	Guil-Guerrero et al. (2017)
	*Cynoglossum cheirifolium*	9–25	1–13	Gazem et al. (2014)
	*Echium horridum*	17.9–20.1	3.4–8.9	Guil‐Guerrero et al. (2006)
	*Echium humile*	6.6–10.3	7.9–10.9	Guil-Guerrero et al. (2001)
	*Echium italicum*	26	7.6	Erdemoglu et al. (2004)
	*Echium plantagineum*	18–23	2.2–9.3	Erdemoglu et al. (2004)
	*Myosotis arvensis*	41.5	1.7	Guil-Guerrero et al. (2003)
	*Myosotis nemorosa*	19.9	20.23	Guil-Guerrero et al. (2001)
	*Myosotis soleirolii*	25.5	4.4	Guil-Guerrero et al. (2017)
Grossulariaceae	*Ribes nigrum* (black currant)	7.6–22.3	11.6–22.7	Ruiz Del Castillo et al. (2004), Goffman and Galletti (2001)
	*Ribes rubrum* (red currant)	6.3–22.4	3.3–7.9	Golovenko et al. (2021)
	*Ribes aureum* (golden currant)	8.3–12.0	4.9–8.6	Lyashenko et al. (2019)
	*Ribes alpinum* (alpine currant)	8.9–19.3	4.5–15.3	Golovenko et al. (2021), Lyashenko et al. (2019)
	*Ribes uva-crispa* L. (gooseberry)	18–20	8–12	Piskernik et al. (2018)

Despite their commercial dominance, plant seeds are inherently limited by low GLA titers, failing to satisfy escalating market demands. Furthermore, enriching these low-concentration oils necessitates rigorous downstream fractionation, a process that incurs prohibitive production costs (Liu et al. 2023). These economic and technical constraints underscore the necessity of developing alternative, high-yielding biosynthetic platforms.

### Microbial production hosts

To circumvent the agronomic constraints of plant-derived oils (Rizzo et al. 2023), microbial fermentation has emerged as an attractive alternative platform. Compared with agricultural production, microbial systems offer shorter production cycles, greater process controllability, and the potential for scalable cultivation (Gupta et al. 2021). Consequently, research has focused on identifying stable, hyper-producing strains among filamentous fungi, yeasts, microalgae, and cyanobacteria (Zhang et al. 2022). Representative GLA-producing microorganisms are summarized in Table 2.

**Table 2:** Fatty acid profiles of key plants and fungi for GLA production

	Oil content (% w/w)	Relative (% w/w) of major fatty acids									References
		16:0	16:1	18:0	18:1	18:2	18:3 ω-6	18:3 ω-3	20:1	22:1	
*M. circinelloides*	25	22	1	6	40	11	18	–	–	–	Ratledge (2005)
*M. isabellina*	50	27	1	6	44	12	8	–	0.4	–	Dyal et al. (2005)
*M. ramanniana*	40	24	–	5	51	10	10	–	–	–	Dyal et al. (2005)
*Syzygites megalocarpus*	22	14	–	1	12	10	62	–	–	–	Weete et al. (1998)
Evening primrose	16	6	–	2	8	75	8–10	0.2	0.2	–	Ratledge (2005),Farag et al. (2023)
Borage	30	10	–	4	16	40	22	0.5	4.5	2.5	Tanwar et al. (2021)
Black currant	30	6	–	1	10	48	17	13	–	–	Traifler et al. (1988)

#### Filamentous fungi for GLA production

In 1948, Bernhard et al. identified an octadecatrienoic acid from *Phycomyces blakesleeanus* as structurally identical to GLA, providing early evidence that filamentous fungi could serve as microbial sources of GLA (Bernhard and Albrecht 1948). Subsequent studies further revealed that GLA production is widely distributed among several oleaginous filamentous fungi, particularly members of Mucorales, which subsequently became major targets for the screening of high-producing strains (Shaw 1966, 1967). 

Colin Ratledge conducted an extensive screening of over 300 species and strains across 13 genera for GLA production over 6 years. The study identified *Cunninghamella*, *Mucor*, and *Rhizopus* as the most promising oleaginous candidates. Despite significant intraspecific variability in lipid accumulation, as exemplified by *M. circinelloides* with lipid contents of 3–37% and GLA fractions of 8–32%, these genera showed considerable potential. Their fermentation profiles (summarized in Table 3) subsequently established them as the primary candidates for industrial scale-up.

The transition to industrial-scale production was pioneered by Idemitsu Kosan Co., Ltd. (Japan). This advancement was supported by early studies published by Suzuki in 1981, who identified *M. isabellina* as a highly prolific strain capable of reaching a total lipid content of 86.1% (Suzuki et al. 1981). However, the final GLA content is only about 10% of the total fatty acids (Conti et al. 2001). This concentration was insufficient to economically compete with established plant sources, such as borage oil (~ 23% GLA), thereby limiting its market viability. To overcome this bottleneck, optimization efforts shifted toward *M. circinelloides*. Consequently, Idemitsu successfully commercialized the world’s first microbial GLA nutritional supplement, achieving a competitive GLA content of 28.3%.

**Table 3:** GLA content of selected fungal species

Family	Genus and species	Total lipid content (%)	GLA content of total fatty acids (%)	References
Cunninghamellaceae	*Cunninghamella blakesleeana*	23–36	7–21	Sukrutha et al. (2014)
	*Cunninghamella echinulata*	18–37	10–24	Kosa et al. (2018), Fakas et al. (2006)
	*Cunninghamella elegans*	13-28.5	21	Vasilakis et al. (2024)
Mortierellaceae	*Mortierella isabellina*	26–30	5.9–13.9	Fakas et al. (2009), Papanikolao and Aggeli (2019)
	*Mortierella ramanniana*	40	10	Ratledge (2005)
Mucoraceae	*Mucor circinelloides*	3-41.6	8-27.1	Zhang et al. (2017), Tang et al. (2015)
	*Mucor genevensis*	2–27	11–32	Ratledge (2005)
	*Mucor mucedo*	10–28	1-28.4	Ratledge (2005)
	*Mucor racemosus*	3–32	0–14	Calarnou et al. (2024)
	*Mucor fragilis*	30–32	24.5	Kosa et al. (2018)
	*Mucor rouxii*	11.6–18.9	9–25	Lindberg and Hansson (1991)
Rhizopodaceae	*Rhizopus microsporus*	13.1	6.0-18.8	Kosa et al. (2018)
	*Rhizopus stolonifer*	6–11	8–28	Ratledge (2005)

#### Yeasts for GLA production

Yeasts are attractive microbial lipid production hosts because of their robust growth, lipid accumulation capacity, and well-established genetic manipulation systems. However, wild-type oleaginous yeasts generally do not accumulate substantial amounts of GLA because the complete desaturation pathway required for GLA biosynthesis is absent or insufficient (Wang et al. 2023). For example, *Yarrowia lipolytica* preferentially accumulates saturated fatty acids, particularly stearic acid, resulting in a lipid profile characterized by high stearic acid and relatively low levels of unsaturated fatty acids (Naveira-Pazos et al. 2023).

To overcome this metabolic limitation, *S. cerevisiae*, *Lipomyces kononenkoae*, and *Y. lipolytica* have been explored as heterologous hosts for GLA production (Ma et al. 2020, Cao et al. 2022, Gu et al. 2023). Engineered *L. kononenkoae* accumulated GLA at approximately 1.2% of total fatty acids (Wang et al. 2011), whereas a recombinant *Y. lipolytica* strain achieved a GLA titer of 71.6 mg/L (Sun et al. 2017).

Despite these advances, the GLA production levels reported for engineered yeasts remain lower than those achieved by several natural fungal producers. Nevertheless, their genetic tractability makes yeasts valuable platforms for pathway reconstruction and further strain engineering.

#### Microalgae and cyanobacteria for GLA production

Cyanobacteria, particularly *Arthrospira* species (commercially known as *Spirulina*), represent a significant alternative reservoir of GLA. In a comprehensive screening of 19 strains, Cohen et al. (1987) identified GLA in 18 isolates, with concentrations ranging from 30 to 35% of total fatty acids, and *Arthrospira platensis* exhibited particularly high GLA accumulation. Beyond *Spirulina*, GLA accumulation has also been reported in eukaryotic microalgae, including *Chlorella vulgaris* and the marine haptophyte *Isochrysis galbana* (Ahamefule et al. 2023).

Despite their relatively high GLA fractions, microalgal and cyanobacterial platforms generally exhibit lower total lipid productivity and biomass accumulation than several oleaginous fungal systems (Latifi et al. 2025). Consequently, they have not yet replaced fungal or plant sources as the major platforms for bulk oil production. At present, *Spirulina*-based products more commonly exploit GLA as a nutritional component rather than as the sole target product. Among the available microbial hosts, Mucorales remain particularly attractive for GLA production because of their lipid accumulation capacity, fermentation performance, and relatively high GLA-producing potential (Fazili et al. 2022).

## Biosynthetic pathways of GLA

Microbial GLA biosynthesis proceeds through the formation of C18 fatty acid precursors followed by sequential desaturation reactions. De novo fatty acid synthesis first generates saturated C16 and C18 fatty acids, which are subsequently converted to oleic acid and LA through desaturation. LA then serves as the immediate precursor of GLA and is converted to GLA by Δ6-desaturase. Therefore, both precursor fatty acid formation and the desaturation steps leading from oleic acid to LA and ultimately to GLA are central to microbial GLA biosynthesis (Nandoskar et al. 2026, Guo et al. 2022, Zhuang et al. 2022).

### Formation of fatty acid precursors

The pathway initiates with glucose uptake, mediated by membrane transporters via facilitated diffusion (Navale 2024). In the cytosol, glycolysis catabolizes glucose into two molecules of pyruvate, generating adenosine triphosphate (ATP) and reduced nicotinamide adenine dinucleotide (NADH) (Chandel and Glycolysis 2021). Subsequently, under the action of pyruvate transport proteins, pyruvate is transported into the mitochondria via specific transport systems (Zangari et al. 2020). Inside the mitochondrial matrix, with the collaborative catalysis of pyruvate dehydrogenase (PDH), pyruvate loses one carbon molecule and binds with coenzyme A to form acetyl-CoA (Casanova et al. 2023). Pyruvate is also carboxylated by pyruvate carboxylase (PC) to generate oxaloacetate, thereby replenishing the oxaloacetate pool required for continuous operation of the tricarboxylic acid (TCA) cycle (Yuan et al. 2022).

**Fig. 3:**
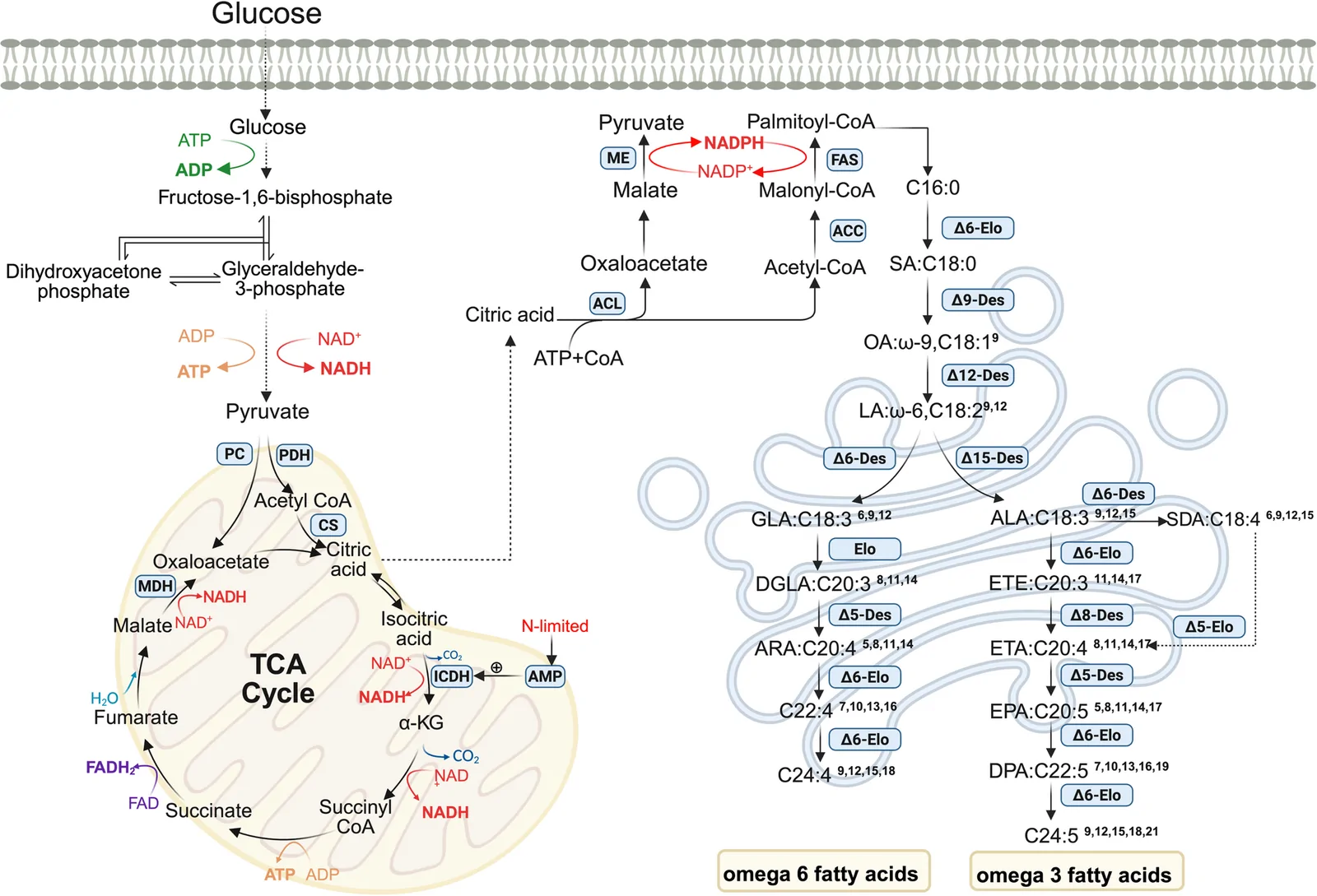
Fatty acid biosynthesis pathway in filamentous fungi. ACC, acetyl-CoA carboxylase; ACL, ATP citrate lyase; ADP, adenosine diphosphate; ALA, α-linolenic acid; AMP, adenosine monophosphate; ARA, arachidonic acid; ATP, adenosine triphosphate; C16:0, palmitic acid; C18:0, stearic acid; CoA, coenzyme A; CS, citrate synthase; DGLA, dihomo-γ-linolenic acid; DPA, docosapentaenoic acid; Elo, elongase; EPA, eicosapentaenoic acid; ETA, eicosatetraenoic acid; ETE, eicosatrienoic acid; FAD, flavin adenine dinucleotide; FADH₂, reduced flavin adenine dinucleotide; FAS, fatty acid synthase; GLA, γ-linolenic acid; ICDH, isocitrate dehydrogenase; LA, linoleic acid; MDH, malate dehydrogenase; ME, malic enzyme; NAD⁺, nicotinamide adenine dinucleotide; NADH, reduced nicotinamide adenine dinucleotide; NADP⁺, nicotinamide adenine dinucleotide phosphate; NADPH, reduced nicotinamide adenine dinucleotide phosphate; OA, oleic acid; PC, pyruvate carboxylase; PDH, pyruvate dehydrogenase; SDA, stearidonic acid; TCA, tricarboxylic acid cycle; α-KG, α-ketoglutarate; Δ5-Des, Δ5-desaturase; Δ6-Des, Δ6-desaturase; Δ8-Des, Δ8-desaturase; Δ9-Des, Δ9-desaturase; Δ12-Des, Δ12-desaturase; Δ15-Des, Δ15-desaturase; Δ5-Elo, Δ5-elongase; Δ6-Elo, Δ6-elongase

Lipid biosynthesis in oleaginous fungi is a highly compartmentalized process involving the cytoplasm, mitochondria, and endoplasmic reticulum (Fig. 3). Carbon substrates such as glucose are first metabolized through glycolysis to generate pyruvate, which enters mitochondrial metabolism and contributes to the formation of acetyl-CoA (Navale 2024, Chandel and Glycolysis 2021, Zangari et al. 2020, Casanova et al. 2023, Yuan et al. 2022). In oleaginous fungi, citrate can subsequently be exported from the mitochondria to the cytosol, thereby supplying cytosolic acetyl-CoA for fatty acid biosynthesis (Wang et al. 2024). Meanwhile, reduced nicotinamide adenine dinucleotide phosphate (NADPH) provides the reducing power required for fatty acid synthesis (Dutta et al. 2025, Wu et al. 2024).

Cytosolic acetyl-CoA is carboxylated by acetyl-CoA carboxylase (ACC) to form malonyl-CoA (Liao et al. 2022). Fatty acid synthase (FAS) then utilizes acetyl-CoA, malonyl-CoA, and NADPH for iterative chain elongation, producing predominantly palmitic acid (C16:0) and stearic acid (C18:0) (Nandoskar et al. 2026) . These saturated fatty acids subsequently enter the elongation-desaturation pathway associated with the endoplasmic reticulum, where a series of elongases and desaturases generates unsaturated fatty acids and PUFAs (Guo et al. 2022, Zhuang et al. 2022).

For GLA biosynthesis, the desaturation reactions involving C18 fatty acids are particularly important. Stearic acid is first desaturated by Δ9-desaturase to form oleic acid, which is subsequently converted to LA by Δ12-desaturase. LA then serves as the direct precursor for GLA formation and is further desaturated by Δ6-desaturase to generate GLA (Zhuang et al. 2022). Therefore, the formation of LA and its subsequent Δ6-desaturation constitute the key GLA-specific steps within the broader fungal fatty acid biosynthetic network.

### GLA biosynthesis in microalgae

Distinct from the predominantly heterotrophic metabolism of oleaginous fungi, fatty acid biosynthesis in microalgae is closely coupled to photosynthetic carbon fixation and is highly compartmentalized (Muñoz et al. 2021). Carbon fixed through the Calvin-Benson cycle is converted into glyceraldehyde-3-phosphate (GAP), pyruvate, and subsequently acetyl-CoA, which serves as the principal precursor for de novo fatty acid synthesis (Hao et al. 2023, Behera et al. 2021, Li et al. 2023).

In the chloroplast, a prokaryotic-type II fatty acid synthase (FAS II) system utilizes acetyl-CoA and malonyl-CoA to generate predominantly C16 and C18 fatty acids (Magnuson et al. 1993). These fatty acids can subsequently be exported from the chloroplast and further modified through elongation and desaturation reactions (Sun et al. 2019).

**Fig. 4:**
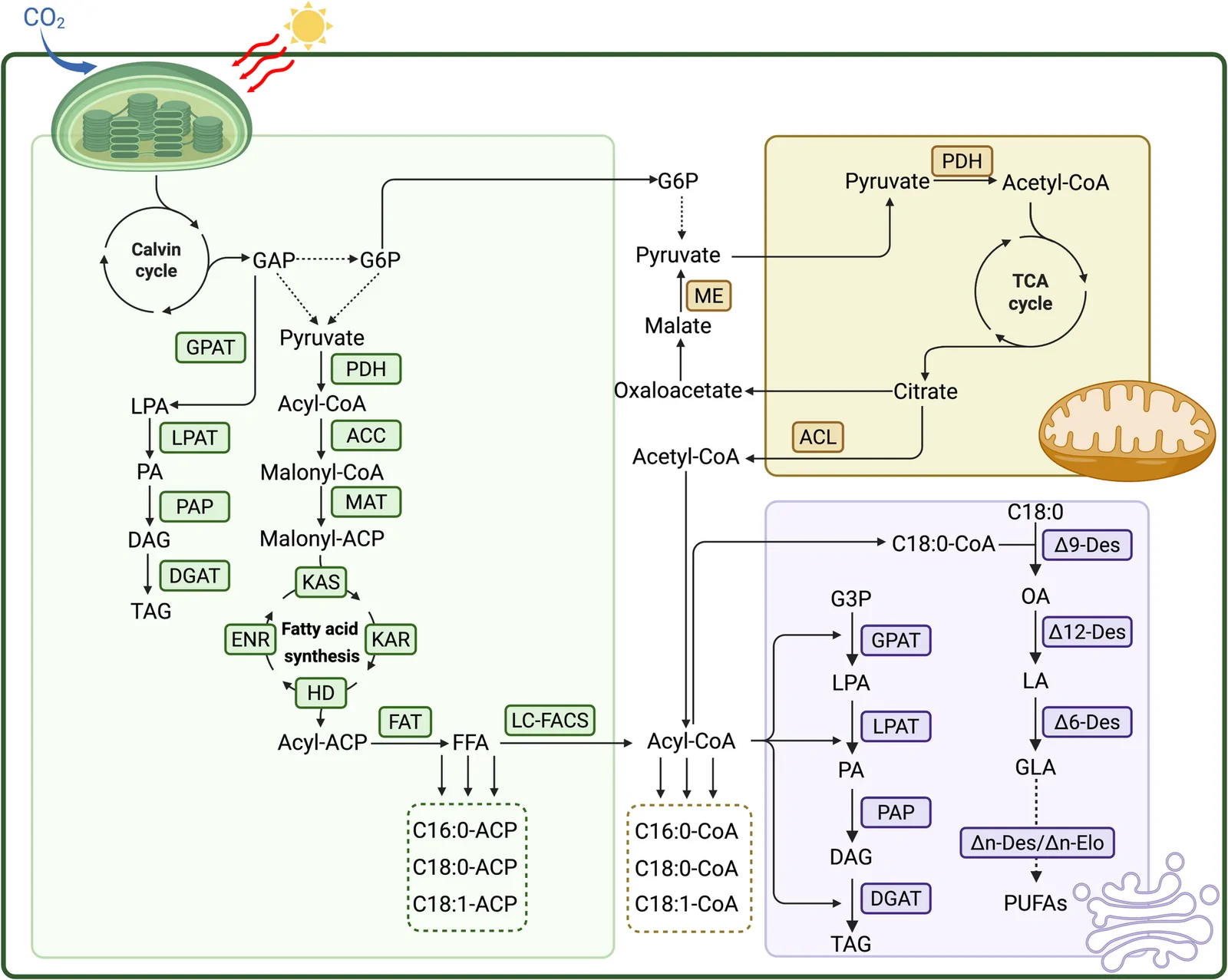
Overview of fatty acid and GLA biosynthesis in microalgae. ACC, acetyl-CoA carboxylase; ACP, acyl carrier protein; ACL, ATP citrate lyase; CoA, coenzyme A; DAG, diacylglycerol; DGAT, diacylglycerol acyltransferase; ENR, enoyl-ACP reductase; FAT, fatty acyl-ACP thioesterase; FFA, free fatty acid; GAP, glyceraldehyde-3-phosphate; G3P, glycerol-3-phosphate; G6P, glucose-6-phosphate; GLA, γ-linolenic acid; GPAT, glycerol-3-phosphate acyltransferase; HD, 3-hydroxyacyl-ACP dehydratase; KAR, 3-ketoacyl-ACP reductase; KAS, 3-ketoacyl-ACP synthase; LA, linoleic acid; LC-FACS, long-chain fatty acyl-CoA synthetase; LPA, lysophosphatidic acid; LPAT, lysophosphatidic acid acyltransferase; MAT, malonyl-CoA: ACP transacylase; ME, malic enzyme; OA, oleic acid; PA, phosphatidic acid; PAP, phosphatidic acid phosphatase; PDH, pyruvate dehydrogenase; PUFAs, polyunsaturated fatty acids; TAG, triacylglycerol; TCA, tricarboxylic acid cycle; Δ6-Des, Δ6-desaturase; Δ9-Des, Δ9-desaturase; Δ12-Des, Δ12-desaturase; Δn-Des, Δn-desaturase; Δn-Elo, Δn-elongase

The formation of GLA depends on the sequential desaturation of C18 fatty acid precursors. Stearic acid is first converted to oleic acid through Δ9-desaturation, followed by Δ12-desaturation to generate LA. LA then serves as the direct precursor of GLA and undergoes Δ6-desaturation to form GLA. As illustrated in Fig. 4, these reactions occur downstream of de novo fatty acid synthesis and are integrated with intracellular lipid trafficking and membrane-lipid metabolism (Muñoz et al. 2021, Sun et al. 2019). Therefore, the availability of LA and the activity of the Δ6-desaturation step are central to GLA formation in microalgal systems.

In addition to PUFA formation, a portion of the newly synthesized fatty acids is incorporated into glycerolipids through the Kennedy pathway and ultimately stored as triacylglycerols (TAGs) (Magnuson et al. 1993). Consequently, the distribution of fatty acids between membrane lipids, storage lipids, and further desaturation pathways can influence the final GLA content and overall lipid composition. This compartmentalized organization distinguishes microalgal GLA biosynthesis from that of heterotrophic filamentous fungi and provides multiple metabolic nodes for subsequent strain and process optimization.

## Strategies to enhance GLA accumulation in oleaginous microorganisms

**Fig. 5:**
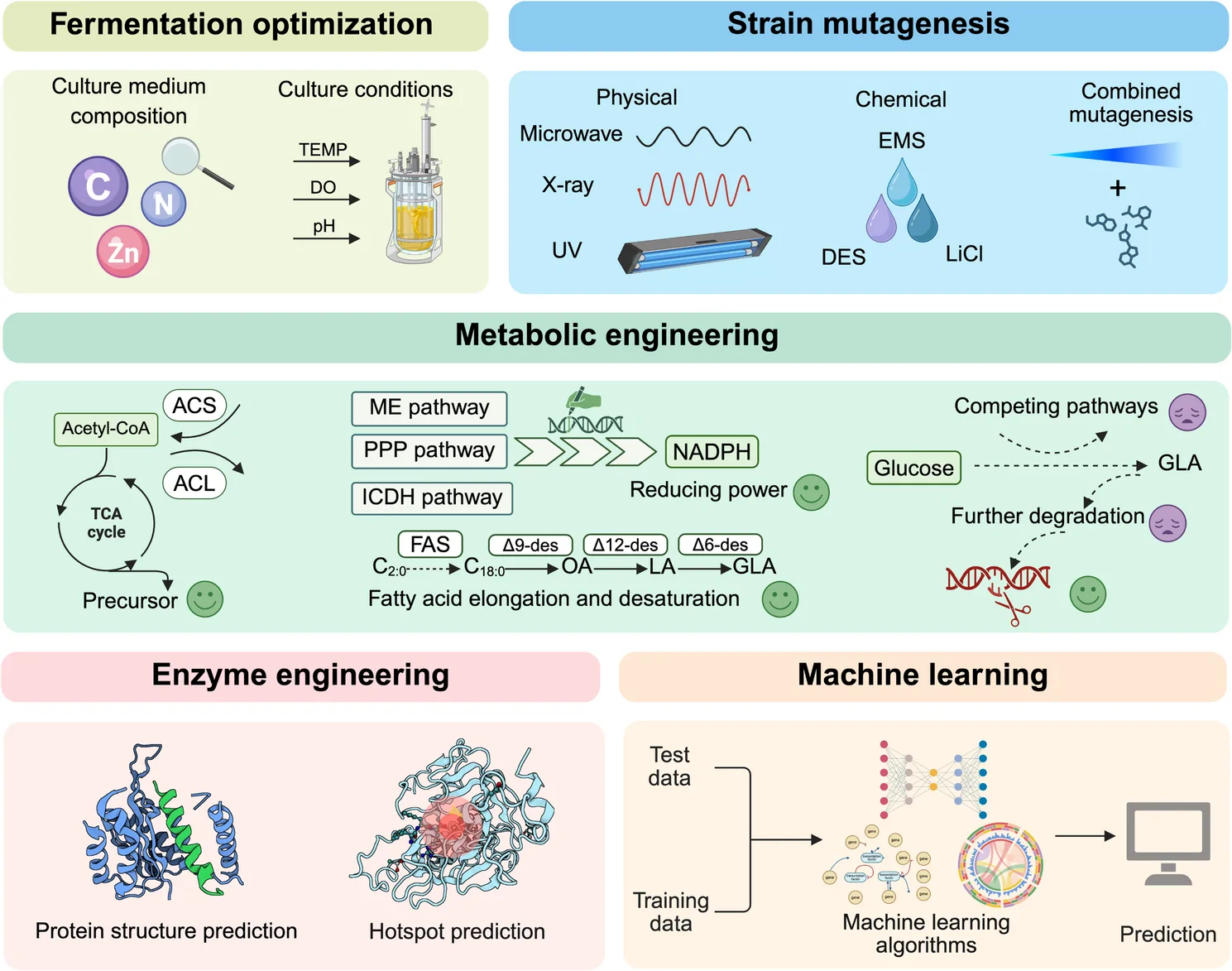
Strategies to enhance GLA accumulation in oleaginous microorganisms. TEMP, temperature; DO, dissolved oxygen; EMS, ethyl methanesulfonate; DES, diethyl sulfate; LiCl, lithium chloride; ACS, acetyl-CoA synthetase; ACL, ATP citrate lyase; TCA, tricarboxylic acid cycle; ME, malic enzyme; PPP, pentose phosphate pathway; ICDH, isocitrate dehydrogenase; NADPH, reduced nicotinamide adenine dinucleotide phosphate; FAS, fatty acid synthase; OA, oleic acid; LA, linoleic acid; GLA, γ-linolenic acid; Δ9-Des, Δ9-desaturase; Δ12-Des, Δ12-desaturase; Δ6-Des, Δ6-desaturase

The biosynthetic characteristics described above indicate that microbial GLA accumulation depends on both the supply of fatty acid precursors and the efficiency of the subsequent desaturation steps. Accordingly, improvement strategies have been developed at multiple levels, including fermentation optimization, strain mutagenesis, metabolic and enzyme engineering, and data-driven strain and process optimization (Fig. 5). These approaches aim to enhance overall lipid accumulation while directing a greater proportion of metabolic flux toward GLA biosynthesis.

### Fermentation process optimization

#### Optimization of medium composition

Nutrient availability is a major determinant of microbial growth and lipid accumulation. In oleaginous microorganisms, lipid accumulation is commonly associated with nutrient imbalance, particularly a high carbon-to-nitrogen (C/N) ratio (Hassane et al. 2024). When nitrogen becomes limiting while excess carbon remains available, cellular metabolism shifts from biomass formation toward lipid accumulation (Babu et al. 2022, Singh et al. 2024, Gong et al. 2026). Therefore, both carbon and nitrogen supply, as well as their relative proportions, represent key parameters for optimizing microbial lipid and GLA production.

Glucose is one of the most widely used carbon sources for oleaginous fungi, although various alternative substrates, including xylose, starch, glycerol, lignocellulosic hydrolysates, and agro-industrial wastes, can also support lipid production in specific strains (Lei et al. 2024). *Mucor* species can utilize a broad range of carbon sources, providing flexibility for the conversion of inexpensive feedstocks into microbial lipids (Zhang et al. 2023). In addition to purified sugars, low-cost substrates such as industrial wastewater (Qiao et al. 2022), lignocellulosic biomass (Zhang et al. 2024), and their hydrolysates (Ivančić Šantek et al. 2021), and glycerol (Vasilakis et al. 2024) have also been investigated for microbial lipid and GLA production.

The type and concentration of nitrogen sources further influence lipid accumulation. Dzurendova et al. demonstrated that nitrogen source composition markedly affected biomass formation and lipid production in oleaginous *Mucoromycota* fungi (Dzurendova et al. 2020), while excessive nitrogen availability generally suppressed lipid accumulation (Mohamed et al. 2022). Different nitrogen sources can also alter the activities of lipogenic and NADPH-generating enzymes. For example, organic nitrogen sources have been associated with enhanced ACC and malic enzyme (ME) activities, whereas NH_4_^+^, NaNO_3_, and other inorganic nitrogen sources can differentially affect ATP citrate lyase (ACL), glucose-6-phosphate dehydrogenase (G6PD), 6-phosphogluconate dehydrogenase (6PGD), and isocitrate dehydrogenase (ICDH) activities (Certik et al. 1999). These observations highlight the importance of jointly optimizing nitrogen source composition and C/N ratio rather than considering nitrogen concentration alone.

Beyond macronutrients, inorganic elements are also essential for microbial growth and play a critical role in regulating the pH of the culture medium, thereby creating an optimal environment for microbial proliferation. Muhid et al. (2008) discovered that when the concentration of Zn^2+^ was appropriately increased, the GLA content in *Cunninghamella* sp. increased by 51.23%. Therefore, in the fermentation process of filamentous fungi, it is essential to optimize the types and concentrations of various nutritional elements to develop an optimized culture medium composition, thereby achieving the optimal lipid accumulation effect.

#### Optimization of cultivation conditions and fermentation modes

In addition to the important influence of the nutrient composition on the final GLA production capacity, cultivation conditions, including temperature and dissolved oxygen (DO), as well as fermentation modes such as batch, continuous, and fed-batch cultivation, also affect GLA production.

Researchers have found that temperature significantly influences the degree of fatty acid unsaturation, with lower temperatures generally favoring the accumulation of unsaturated fatty acids (Mhlongo et al. 2021). Additionally, the optimal temperatures for the accumulation of oils and GLA typically differ (Li et al. 2019). During the fatty acid accumulation phase, a decrease in temperature correlates with an increase in lipid unsaturation.

To reconcile this divergence, a two-stage temperature shift strategy was implemented to optimize biomass growth and GLA production. This protocol typically utilizes an initial high-temperature phase to promote biomass growth, followed by a cooling phase to induce GLA synthesis (Song et al. 2022). For instance, Sun et al. (2017) employed a two-stage temperature culture strategy, initially culturing at 28 °C and then at 20 °C for 6 days. This resulted in optimal levels of dry cell weight (DCW), lipid content, and GLA concentration in the recombinant strain, reaching 18.55 g/L, 1.16 g/L, and 71.6 mg/L, respectively. These DCW, lipid, and GLA values were 25.7%, 19.6%, and 60.9% higher, respectively, than those obtained in the control culture experiment conducted at a constant temperature of 28 °C.

In photosynthetic GLA producers, light regime and oxidative conditions represent additional cultivation variables that can influence GLA accumulation. Mofeed et al. reported that cultivating *A. platensis* under continuous illumination and 2 mM H₂O₂ stress achieved a GLA yield of 31.62 mg/g, corresponding to 27.42% of total fatty acids (Mofeed 2019).

Given that desaturases are oxygen-dependent enzymes, DO availability is an important factor affecting PUFA biosynthesis. Many researchers have observed that most oleaginous strains exhibit enhanced lipid accumulation in high-oxygen environments (Chang et al. 2022). However, this does not imply that DO levels should be indiscriminately increased, as they also influence the morphological characteristics of the strains (Zhang et al. 2019). To prevent the growth of strains from being restricted due to insufficient DO, stirring at an appropriate speed or aeration is usually used to increase the DO in practical industrial production (Guerreiro et al. 2019). Furthermore, advanced strategies such as multi-stage DO control and the introduction of oxygen vectors have proven effective in boosting yields. Ren et al. (2010) implemented a dynamic aeration strategy: cycling the aeration rate between 0.4 and 0.6 vvm (volume of air per volume of medium per minute) based on fermentation stages. This protocol optimized the trade-off between biomass growth and lipid synthesis, achieving a high cell density (71 g/L) and a significant 11.21% improvement in docosahexaenoic acid (DHA) productivity (119 mg/L·h) compared to constant aeration controls.

Enhancing the lipid-producing capabilities of microbial host strains will accelerate the development of microbial GLA production. When the intrinsic metabolic potential of these superior strains is synergized with advanced fermentation process control, the techno-economic efficiency of microbial biorefineries is substantially maximized. While fermentation optimization constitutes the current backbone of industrial research, the future paradigm lies in the holistic integration of advanced molecular biology techniques with bioprocess engineering. This convergent approach is poised to maximize microbial GLA titers, thereby establishing a robust, sustainable supply chain independent of agricultural fluctuations.

### Strain mutagenesis

Physical mutagenesis, utilizing agents such as ultraviolet (UV) radiation, X-rays, ion beams, and microwaves, remains a cornerstone of industrial strain improvement (Al-Hawash et al. 2018). Among these, UV irradiation is the most prevalent technique due to its operational simplicity. Mechanistically, UV exposure induces the formation of cyclobutane pyrimidine dimers in DNA, and the subsequent error-prone repair of these lesions introduces random point mutations (Kilbey et al. 1978). For instance, UV mutagenesis applied to *Tetraselmis suecica* successfully elevated lipid production by 1.2–1.4 times under thermal stress conditions (Lo et al. 2022).

Complementary to physical methods, chemical mutagenesis employs alkylating agents like ethyl methanesulfonate (EMS) (Orgel 1965), diethyl sulfate (Yang et al. 2018), or stress agents such as lithium chloride (LiCl) (Suryadi et al. 2022) to induce genomic instability. Demonstrating this approach, Guo et al. achieved a 30–40% increase in lipid accumulation in *Rhodosporidium toruloides* through selection for LiCl and ethanol-H_2_O_2_ tolerance (Guo et al. 2019).

Furthermore, combined or sequential mutagenesis is frequently employed to enhance mutagenic efficiency and expand mutant diversity. Sarayloo et al. implemented a sequential strategy by subjecting a UV-generated *C. vulgaris* mutant to subsequent EMS treatment. This dual protocol yielded a hyper-producing strain exhibiting a 67% increase in biomass and a 35% enhancement in lipid yield relative to the wild-type strain (Sarayloo et al. 2018).

However, strains derived from physical or chemical mutagenesis often exhibit genetic instability and deleterious background mutations, necessitating labor-intensive, time-consuming screening processes to isolate candidates that meet rigorous industrial standards (Zhang et al. 2018). Consequently, to overcome these limitations, classical mutagenesis is increasingly coupled with rational genetic engineering. This synergistic approach allows for the precise optimization of metabolic nodes, thereby maximizing overall strain performance (Chang et al. 2021).

### Metabolic engineering of lipid and GLA biosynthesis

With the progressive elucidation of lipid biosynthetic pathways, rational metabolic engineering has emerged as the premier strategy for constructing high-yield GLA-producing cell factories. As illustrated in Fig. 5, optimizing GLA flux requires a dual approach: upregulating these biosynthetic nodes while simultaneously attenuating competitive pathways to minimize carbon diversion. Recent advancements in the targeted engineering of GLA-producing strains have primarily focused on the regulation of these critical enzymes involved in de novo fatty acid synthesis.

#### Enhancing acetyl-CoA supply

Since cytosolic acetyl-CoA serves as the primary carbon skeleton for fatty acid synthesis (Yoshii et al. 2015, Chen et al. 2013), engineering strategies focus on expanding this intracellular pool through two distinct mechanisms:

- *Reinforcing the Citrate Shuttle* ACL is a key enzyme controlling cytosolic acetyl-CoA availability. Homologous overexpression of ACL markedly enhances fatty acid content, whereas its knockdown significantly reduces lipid accumulation (Chen et al. 2014). Upstream of ACL, the citrate/malate translocase imposes an additional flux bottleneck by controlling citrate export from mitochondria. In *M. circinelloides* WJ11, overexpression of two citrate transporter genes increased lipid accumulation by 44% and 68%, respectively (Yang et al. 2020).
- *Amplifying the Acetyl-CoA Synthetase (ACS) Bypass Pathway *Alternatively, ACS offers a citrate-independent route by directly catalyzing the ligation of acetate and coenzyme A. This pathway circumvents the conventional citrate and ACL-dependent mechanism, enabling de novo lipid biosynthesis, particularly in organisms capable of directly metabolizing acetate as a carbon source (Howard et al. 1974). ACS activity exerts a substantial influence on lipid production, although its relatively low catalytic efficiency and high energy demand can constitute a bottleneck in cytosolic fatty acid synthesis (Krivoruchko et al. 2015). In previous studies, the overexpression of ACS consistently facilitated enhanced carbon flux toward lipid biosynthesis, increased biomass yield, and significantly augmented fatty acid or TAG accumulation, achieving increases of 29.9% in biomass, 11.3% in lipid yields, and a 2.4-fold enhancement in TAG accumulation, respectively (Lin et al. 2006, Yan et al. 2013, Rengel et al. 2018). These results establish ACS as a crucial regulatory target for the optimization of lipid metabolic pathways in engineered strains.

#### Enhancing NADPH supply

The biosynthesis of fatty acids is essentially a reduction process that requires a large input of NADPH. Stoichiometrically, each two-carbon elongation cycle consumes two NADPH molecules, while every subsequent desaturation step requires an additional NADPH equivalent. To sustain this high demand, oleaginous microorganisms rely on multiple NADPH-generating systems, among which the ME pathway and pentose phosphate pathway (PPP) have received particular attention in lipid biosynthesis (Ratledge 2014).

ME has long been regarded as the major source of reductive power for lipogenesis in oleaginous fungi. Overexpression of ME in *M. circinelloides* resulted in a 2.5-fold increase in lipid content (Zhang et al. 2007), and isoform-specific overexpression (*malC* and *malD*) enhanced GLA yield by 16.73% and 46.76%, respectively (Fazili et al. 2022).

While ME has traditionally been viewed as the primary NADPH generator, the PPP serves as a critical alternative source, particularly for converting excess glucose flux into reductive power (Wasylenko et al. 2015). The pathway’s flux is controlled by two rate-limiting dehydrogenases: G6PD and 6PGD (Ratledge 2014). Genetic manipulation of these nodes has proven effective. For instance, Jiao et al. achieved a 2.7-fold increase in lipid content (reaching 55.7% of dry cell weight) in *Phaeodactylum tricornutum* via PPP regulation (Xue et al. 2017). Similarly, the overexpression of both G6PD and 6PGD in *Fistulifera solaris* significantly elevated intracellular NADPH levels, driving enhanced lipid accumulation (Osada et al. 2017).

Furthermore, distinct NADPH sources appear to fuel specific metabolic modules. In *M. alpina*, Hao et al. delineated these functional differences, revealing that G6PD primarily sustains fatty acid synthesis, whereas ME is specifically critical for the high-demand desaturation process (GLA formation) (Hao et al. 2016). These findings confirm that the precise manipulation of specific NADPH pools offers a potent strategy for enhancing both lipid accumulation and GLA biosynthesis (Qiao et al. 2017).

#### Enhancing fatty acid elongation and desaturation

Building on the GLA biosynthetic pathway described in Sect.  4.1, GLA production can be further enhanced by engineering the desaturase cascade. Δ6-desaturase, which catalyzes the committed step converting LA to GLA, is the primary rate-limiting target. Since its first isolation from *Synechocystis* sp., heterologous expression of Δ6-desaturase has been widely deployed to enhance GLA production across diverse hosts (Reddy et al. 1993).

Combinatorial overexpression strategies have proven particularly effective. For instance, Wang et al. achieved a 1.9-fold increase in GLA titer in *M. circinelloides* by co-overexpressing Δ6-, Δ9-, Δ12-desaturases (Wang et al. 2022). For the synthesis of PUFAs longer than 18 carbons, additional desaturases, such as Δ5- and Δ15-desaturases, and elongation enzymes are required to optimize the pathway.

#### Blocking competing pathways and minimizing lipid turnover

From a systems biology perspective, maximizing lipid titers requires not only the upregulation of biosynthetic enzymes but also the systematic attenuation of competitive metabolic branches via rational metabolic engineering.

- *Ablation of competitive carbon sinks* A primary strategy involves disrupting the synthesis of non-lipid storage compounds, particularly polysaccharides (Jia et al. 2022). For instance, Bhutada et al. reported a 60% increase in TAG content in *Y. lipolytica* following the deletion of *YlGSY1* (glycogen synthase). Similarly, targeted disruption of starch or cyanophycin biosynthesis genes (e.g., *glgC*, *cphA*) in *Synechocystis* sp. elevated fatty acid levels by 24% (Yoo et al. 2023). In eukaryotic microalgae, Chang et al. employed CRISPR-Cas9 to disrupt ADP-glucose pyrophosphorylase (AGP) in *Tetraselmis* sp., yielding a 2.7- to 3.1-fold increase in lipid content (Chang et al. 2020). In fungal systems like *M. alpina*, Chen et al. demonstrated that knocking down competing carbohydrate pathway of *MaTPS* (trehalose-6-phosphate synthase) and *MaPEPCK* (phosphoenolpyruvate carboxykinase) enhanced lipid accumulation by over 20% (Chang et al. 2023).Furthermore, minimizing the secretion of metabolic byproducts is critical. In *Y. lipolytica*, excess carbon is frequently excreted as citric acid rather than converted to lipids (Jia et al. 2022). To address this issue, Sagnak et al. overexpressed the diacylglycerol acyltransferases (*ylDGA1* and *ylDGA2*), effectively redirecting carbon flux from citrate excretion to TAG synthesis and elevating lipid content from 18 to 55% (Sagnak et al. 2018). Furthermore, minimizing the secretion of metabolic byproducts is critical. In *Y. lipolytica*, excess carbon is frequently excreted as citric acid rather than converted to lipids (Jia et al. 2022). To address this issue, Sagnak et al. overexpressed the diacylglycerol acyltransferases (*ylDGA1* and *ylDGA2*), effectively redirecting carbon flux from citrate excretion to TAG synthesis and elevating lipid content from 18 to 55% (Sagnak et al. 2018).
- *Suppression of fatty acid catabolism* Preventing lipid turnover is as critical as enhancing biosynthesis for maximizing final lipid titers. Dulermo et al. achieved lipid yields of 65–75% DCW in *Y. lipolytica* through a combinatorial strategy by overexpressing TAG synthesis genes (*SCT1*, *DGA1*) and simultaneously deleting TAG lipase genes (*TGL3*, *TGL4*) (Dulermo and Nicaud 2011). Similarly, targeting the β-oxidation pathway, Huang et al. employed homologous recombination to selectively knockout three acyl-CoA oxidase genes, which are key catalysts in the initial step of β-oxidation, in *Schizochytrium* sp., revealing a consistent lipid augmentation across all knockout strains (Huang et al. 2021). Furthermore, Imatoukene et al. deleted the *DGA1*, *DGA2*, and *LRO1* genes in the Kennedy pathway alongside the *POX1* gene involved in fatty acid β-oxidation in *Y. lipolytica*, demonstrating that the degradation rate of conjugated LA was significantly reduced in these knockout strains (Imatoukene et al. 2017).

In summary, these investigations underscore the critical role of metabolic engineering in optimizing lipid biosynthesis through a multi-dimensional approach involving the amplification of key precursor availability, the bolstering of NADPH generation, the enhancement of FAS activity, and the systematic suppression of competitive biosynthetic pathways. Such modifications have yielded substantial improvements in lipid accumulation across various microbial systems, presenting a robust framework for the engineering of high-yield strains, particularly for the enhanced biosynthesis of GLA.

### Engineering enzymes for microbial lipid biosynthesis

Enzyme engineering stands as a potent tool for the precise regulation of enzymes, enabling the modulation of their stability, activity, and specificity (Ali et al. 2020). Under industrial conditions, the majority of metabolic enzymes frequently manifest low catalytic activity, diminished stability, and susceptibility to substrate inhibition (Zhou et al. 2024). Hence, the application of enzyme engineering for the precise modification of enzymes, particularly those involved in lipid metabolism, is anticipated to significantly augment lipid accumulation in oleaginous microorganisms. For instance, Hayashi et al. successfully enhanced the fatty acid content of *Schizochytrium* sp. by redesigning the structure and copy number of tandem acyl carrier protein (ACP) domains within the polyketide synthase subunit A (Hayash et al. 2016). Similarly, utilizing an approach that integrates molecular modeling with transcription activator-like effector nucleases technology, Rigouin et al. performed site-directed mutagenesis on the isoleucine residue (I1220) within the fatty acid-binding pocket of the FAS in *Y. lipolytica* (Rigouin et al. 2017). This targeted modification altered chain-length specificity, favoring the synthesis of shorter-chain fatty acids. Furthermore, Feng et al. engineered the fatty acid profile in *E. coli* by modulating the activity of thioesterase (TE), the critical enzyme responsible for chain termination and carbon chain length determination (Feng et al. 2018).

Collectively, these investigations demonstrate the efficacy of enzyme engineering in tailoring both the quantity and composition of microbial lipids. Moving forward, the integration of computational structural biology with rational design is essential. By elucidating the catalytic mechanisms of key GLA-biosynthetic enzymes and predicting mutation hotspots via in silico modeling, researchers can engineer variants with superior kinetics. Although the application of enzyme engineering in lipid biosynthesis is currently in its nascence compared to metabolic pathway engineering, it holds immense potential for unlocking the next generation of GLA hyper-producers as these technologies mature.

### Machine learning and data-driven approaches for strain and bioprocess optimization

As biotechnological datasets expand exponentially, ML has emerged as a transformative data-driven paradigm that utilizes advanced algorithms to decipher complex biological patterns, thereby enhancing predictive accuracy in both strain engineering and bioprocess control (Pradipta Patra et al. 2023). In the realm of systems biology, ML plays a pivotal role in constructing and refining genome-scale metabolic models (GEMs), enabling the simulation of metabolic fluxes to identify non-intuitive gene targets (Yang et al. 2023). For instance, Vongsangnak et al. utilized comparative GEM analysis across *M. circinelloides*, *M. alpina*, and *Y. lipolytica* to pinpoint conserved metabolic pathways and potential engineering targets (Vongsangnak et al. 2016). Similarly, Kim et al. applied GEM-guided simulations to *Y. lipolytica*, validating overexpression targets that resulted in a 45% increase in lipid accumulation (Kim et al. 2019). Beyond pathway prediction, ML also empowers rational enzyme engineering; Greenhalgh et al. employed ML algorithms to navigate protein fitness landscapes, successfully optimizing fatty acyl-ACP reductase activity to modulate fatty acid metabolism (Greenhalgh et al. 2021). Furthermore, flux balance analysis has clarified the critical role of specific enzymes, as demonstrated by Ye et al. (2015), who identified the ME as essential for ARA and NADPH production in *M. alpina*, with ME knockout causing a 38.28% reduction in ARA. The integration of ML techniques into GEM enhances systems biology research, supporting the development of efficient microbial cell factories and driving biotechnological innovation.

Beyond genetic manipulation, ML is increasingly indispensable for optimizing the fermentation process. The fermentation process encompasses intricate mechanisms, wherein even subtle adjustments to the parameters exert a notable influence on the ultimate fermentation outcomes. By leveraging historical process data, ML models can predict optimal fermentation parameters, significantly enhancing operational efficiency (Khaleghi et al. 2021). Over the past decades, a diverse array of algorithms, including linear regression (Michael et al. 2024), Gaussian process (GP) (Yang et al. 2025), gradient enhancement (Khaleghi et al. 2021), Bayesian (Wang et al. 2023), fuzzy logic (Mahanty 2023), artificial neural networks (ANN) (Gbashi et al. 2023), decision trees (Fahimi Bandpey et al. 2024), and principal component analysis (Zhang et al. 2022) has been deployed to model these complex landscapes. For example, Yang et al. successfully employed a GP-driven framework to optimize the bioconversion of corn stover to GLA. By utilizing GP to rigorously model the fermentation parameters (time, glucose, C/N ratio) and enzymatic hydrolysis conditions, they achieved a GLA titer of 2.13 g/L, representing a significant improvement over the 1.82 g/L yield obtained via traditional Response Surface Methodology (RSM). This study exemplifies how ML strategies can surpass conventional statistical tools by providing uncertainty estimates and more accurate predictions for unobserved experimental points. Vimali et al. integrated RSM with ANN to model lipid recovery in *Desmodesmus* sp., establishing a robust computational framework tailored to algal strains (Vimali et al. 2022). Taking process optimization further, Kumar et al. demonstrated the power of coupling ANN with genetic algorithms to simultaneously maximize lipid and biomass titers in *Pichia pastoris*, signifying a substantial advancement in productivity (Kumar et al. 2020). Additionally, Sultana et al. developed a Bayesian optimization combined with a vector regression algorithm to predict the model for microalgal biodiesel production. Upon validation, it was found that this model exhibited a performance improvement of 36.03% over previous studies (Sultana et al. 2022).

Ultimately, the applicability of specific algorithms varies depending on the strain characteristics and data complexity, making the selection of the most suitable computational approach crucial for industrial GLA production. When coupled with high-quality training data, these ML-driven strategies maximize the predictive functionality of computational models, providing a rigorous guide for process optimization. By creating a feedback loop between computational predictions and experimental validation, ML establishes a robust framework for achieving enhanced production yields and driving future biotechnological innovations in microbial lipid manufacturing.

## Downstream processing for cost-effective GLA production

### Cell pretreatment and disruption

The lipid of oleaginous microorganisms is mostly stored within cellular structures or as components of cell membranes. Effective extraction of these lipids necessitates the disruption of the cell membrane (Fabiszewska et al. 2023). Cell disruption plays a critical role in lipid extraction from microbial biomass, as its efficiency directly impacts the effectiveness of subsequent downstream processes (Rollin et al. 2023). Following disruption, lipids are typically recovered via solvent extraction and subjected to refinement to isolate high-purity PUFAs, thereby augmenting their commercial value. However, the techno-economic challenges associated with efficient lipid recovery have largely restricted these processes to the laboratory scale (Huang et al. 2023). The following discussion outlines scalable extraction strategies aimed at bridging this gap.

The selection of an appropriate pretreatment regimen is pivotal for process economics (Khoo et al. 2020). These methods can be broadly categorized into mechanical and non-mechanical based on energy transfer requirements (Gautério et al. 2023). Conventional mechanical pretreatment methods include heat treatment, dry fungal cell disruption using conventional mechanical forces, such as ball mills, high-pressure homogenizers, and vibratory disruptors, as well as non-conventional forces like ultrasonication (Mat Husin et al. 2024), microwave radiation (Kumar et al. 2022), and pulsed electric field (Silve et al. 2024). While these methods offer operational simplicity and high disruption efficiency (Oh et al. 2022), they often require an initial dewatering and drying step, which imposes a prohibitive energy penalty on the overall production process (Gupta et al. 2021). Considering the feasibility of the production process, it is recommended to avoid the energy-intensive drying step and instead utilize the non-mechanical wet extraction method (Huang et al. 2023). These gentler methods are more energy-efficient and are commonly employed for extracting lipids from wet microbial biomass.

Among wet processing methods, enzymatic hydrolysis represents a promising non-mechanical strategy. By eliminating the need for thermal drying, this approach significantly reduces energy consumption and operational costs while preserving the structural integrity of heat-sensitive PUFAs like GLA (Costa et al. 2024). Leveraging high biological specificity, lytic enzymes effectively degrade the complex polysaccharide matrix of fungal cell walls, facilitating the release of cytoplasmic lipids (Liang et al. 2023, Mantri et al. 2025). Furthermore, the efficacy of enzymatic lysis can be amplified through combinatorial strategies, such as coupling with microwave treatment (Guo et al. 2024), ball milling (Alavijeh et al. 2020), or heat treatment (Banerjee et al. 2024), which can further enhance the efficient extraction of microbial lipids. Despite its environmental and quality advantages, enzymatic hydrolysis faces industrial hurdles, particularly the structural heterogeneity of fungal cell walls which complicates enzyme cocktail selection (Huang et al. 2023). Moreover, the high cost of industrial enzymes, prolonged reaction times, and the requirement for precise reaction control currently constitute significant techno-economic barriers to its widespread commercial application.

### Lipid extraction

Following the disruption of cellular barriers, the accessible intracellular lipids must be recovered using appropriate extraction protocols. Solvent extraction constitutes the predominant methodology, exploiting the differential solubility of lipids in organic solvents to isolate them from the aqueous biomass matrix, followed by solvent evaporation to yield crude oil. Advancements in scientific research have led to the development of various extraction techniques for lipids, including Soxhlet extraction (Banerjee et al. 2024), the Folch method (Legodi and Moganedi 2023), the Bligh and Dyer method (Jabinski et al. 2024), supercritical fluid extraction, pressurized fluid extraction, among others.

#### Traditional extraction methods

The shared non-polar nature of organic solvents and lipid molecules significantly enhances their mutual solubility, making organic solvents highly effective for lipid extraction. This method has a long-established history, dating back to 1879 when Soxhlet first introduced solvent-based lipid extraction using ether to isolate milk lipids. Since then, the procedure has remained the most widely recognized and convenient approach worldwide and is frequently employed as a benchmark for evaluating the efficiency of emerging lipid extraction techniques (Ochsenreither et al. 2016). In 1957, Folch developed a widely recognized method for isolating total lipids from animal tissues, employing a chloroform/methanol (2:1) solvent mixture, which continues to serve as a global benchmark for lipid extraction (Folch et al. 1957). Bligh and Dyer later improved this method by creating a faster, more efficient extraction technique (Bligh and Dyer 1959). Despite their efficacy, the substantial consumption of toxic, volatile solvents raises significant environmental and safety concerns, necessitating the search for sustainable alternatives.

The efficiency of extraction is intrinsically linked to solvent polarity and its ability to penetrate the cell wall matrix (Saini et al. 2021). Attempts have been made to replace chlorinated solvents with less toxic alternatives, such as isopropanol/hexane or ethyl acetate/ethanol systems (Banerjee et al. 2024). However, these alternatives were less effective compared to the Folch method. Furthermore, different microorganisms exhibit varying affinities for lipid extraction techniques. For instance, Hussain et al. identified the Bligh and Dyer method as the most effective for extracting lipids from *M. isabellina*, whereas Santos et al. demonstrated that an ultrasound-assisted chloroform/methanol method was optimal for *C. vulgaris* (Santos et al. 2015). However, the high toxicity of conventional solvents has limited their use in large-scale biodiesel production, prompting research into safer, non-volatile, cost-effective alternatives for lipid extraction (Akonjuen et al. 2023).

Certik et al. conducted an extensive evaluation of 17 solvent combinations and concluded that a two-step hexane/ethanol extraction method was optimal for extracting GLA from *M. circinelloides* (Čertík et al. 1996). This method achieved lipid extraction rates comparable to the Folch method, with the added benefit of lower toxicity. Similarly, Cascant et al. analyzed eco-friendly solvents like 2-methyltetrahydrofuran, cyclopentyl methyl ether, and ethyl acetate, identifying ethyl acetate as a lower-toxicity alternative to hexane (Cascant et al. 2017). Conversely, it was reported that while solvents like 2-methyltetrahydrofuran and cyclopentyl methyl ether are sustainable, their extraction efficiencies for microalgal lipids remain inferior to traditional chloroform-based systems (Jesus et al. 2019). While green solvents offer reduced toxicity and environmental impact, their extraction efficiency still lags behind traditional solvents like chloroform/methanol. Thus, further research is urgently needed to identify more effective and environmentally sustainable extraction solvents and techniques.

#### Emerging extraction methods

Deep eutectic solvents composed of hydrogen bond donors and acceptors have emerged as an innovative and environmentally friendly alternative to traditional organic solvents for natural product extraction (Hansen et al. 2021). Characterized by their inherent tunability, biodegradability, and cost-effectiveness, deep eutectic solvent systems can be tailored for high selectivity (Moreno Martínez et al. 2022). In comparative evaluations, Wils et al. demonstrated that deep eutectic solvents outperformed ethyl acetate and dimethyl carbonate in extracting free fatty acids from *Spirulina*, exhibiting superior efficiency and selectivity (Wils et al. 2022). Similarly, Moreno et al. highlighted the efficacy of deep eutectic solvents in recovering total fatty acids and EPA from *Nannochloropsis gaditana*, establishing deep eutectic solvents as a promising green platform for lipid valorization (Moreno Martínez et al. 2022).

Supercritical fluids, which exist between gaseous and liquid states, offer a sustainable alternative to traditional organic solvents due to their high density, solvency, and ability to operate under varying temperatures and pressures (Lee et al. 2021). These fluids enhance selectivity, yield purer extracts, and reduce extraction time (Rodríguez-España et al. 2022). Supercritical CO_2_ is the preferred solvent for lipophilic compounds due to its non-toxicity and mild critical temperature (31 ℃), which preserves heat-sensitive PUFAs like GLA (Tzima et al. 2023). However, pure CO_2_ is non-polar and often requires polar co-solvents to effectively penetrate cell membranes. For instance, Yang et al. (2019) developed a solid matrix-supported supercritical CO₂ extraction approach using diatomite to enhance the recovery of GLA-rich glycolipids from *Arthrospira platensis*, achieving a GLA extraction efficiency of 98% (Yang et al. 2019) . Similarly, Sajilata et al. applied supercritical fluid extraction (SFE) to *Spirulina platensis*, refining the method through response surface optimization to further enhance GLA extraction.

A major limitation of conventional SFE is the requirement for dry biomass, as moisture hinders CO_2_ diffusivity, imposing a high energy penalty for drying (Jesus and Filho 2020). To address this, process intensification strategies such as reactive extraction have been developed. Jafari et al. integrated enzymatic transesterification directly within the SFE process, enabling the direct conversion of wet microalgae into biodiesel, thereby bypassing the energy-intensive drying step (Jafari et al. 2021). Although SFE extraction is regarded as one of the most eco-friendly techniques, challenges remain in scaling it for industrial GLA extraction, particularly due to the precise temperature and pressure demands, as well as GLA’s instability at elevated temperatures.

Pressurized Liquid Extraction (PLE) utilizes liquid solvents at elevated temperatures and pressures to enhance extraction kinetics. Unlike SFE, PLE offers greater solvent flexibility and is particularly advantageous for processing wet biomass, potentially eliminating drying costs (Bhowmick et al. 2024). For example, Castejón et al. used PLE with hexane to achieve a lipid yield of 17.6% from *N. gaditana*, slightly surpassing the 16.9% yield obtained through the conventional Folch method (Castejón and Señoráns 2019). Furthermore, He et al. compared four extraction techniques for *Isochrysis*, including Soxhlet, Folch, and pressurized liquid extraction with n-hexane or ethanol, and found that 90% ethanol yielded the highest lipid extraction rate (He et al. 2019). Single extraction methods often face limitations in lipid recovery. To address this, Jiménez et al. combined SFE and PLE for EPA extraction from *Nannochloropsis* sp. Via a multi-step extraction protocol, the researchers yielded a final extract with a lipid fraction of 85.1%, in which EPA accounted for up to 39.2% (Jiménez Callejón et al. 2022).

Collectively, these emerging technologies offer a paradigm shift toward greener, safer, and potentially more efficient lipid recovery compared to traditional solvent extraction. While laboratory-scale data indicates comparable or superior efficiencies, the industrial viability of these methods depends on rigorous Techno-Economic Analysis (TEA) (Jesus and Filho 2020). Future research must transcend extraction yield alone and integrally assess solvent costs, energy consumption, and equipment maintenance to develop a competitive, scalable framework for commercial GLA production.

## Challenges and future perspectives

The industrialization of GLA production through microbial processes still faces several challenges, with the most critical being the limited GLA production capacity of existing strains. While fermentation and metabolic engineering have shown promise in boosting GLA or lipid accumulation, the genetic recalcitrance of oleaginous microorganisms has hindered the development of advanced gene-editing techniques and genetic manipulation systems for most species. Among native GLA-producing fungi, *M. circinelloides* is particularly amenable to genetic manipulation, with established homologous recombination and CRISPR-Cas9-based genome-editing tools that enable targeted modification of multiple metabolic genes (Wang et al. 2022). In contrast, model yeasts such as *Y. lipolytica* and *S. cerevisiae* offer highly developed genetic toolkits and have been extensively engineered for heterologous PUFA biosynthesis. For example, recent metabolic rewiring of *Y. lipolytica* enabled GLA production at 22.58% of total fatty acids, corresponding to a titer of 386.59 mg/L (Gu et al. 2023).

Future breakthroughs rely on next-generation technologies. The advent of CRISPR-Cas9 enables multiplex genome editing, facilitating the simultaneous modification of competing and biosynthetic pathways to maximize flux. Furthermore, the successful application of ML in fields like enzyme and fermentation engineering has helped address traditional limitations in microbial fermentation. This synergy of biotechnology and artificial intelligence is expected to facilitate the development of superior cell factories.

Another pivotal challenge involves the substitution of high-cost raw materials with economically viable alternatives for the production of GLA, encompassing the utilization of lignocellulosic biomass (Ivančić Šantek et al. 2021), industrial wastewater (Qiao et al. 2022), and similar low-cost resources. Nevertheless, these biowastes may harbor inhibitors that impede microbial growth, necessitating meticulous pretreatment and biologically mediated detoxification of these waste materials. Addressing this challenge, Yang et al. demonstrated an ML-assisted detoxification strategy for corn stover hydrolysates (Yang et al. 2025). By optimizing the removal of inhibitors such as phenols and acetic acid using GP models, they successfully mitigated cell toxicity while reducing the consumption of detoxification agents (activated carbon) by 41%, thereby validating a cost-effective route for utilizing lignocellulosic biomass. Transforming these waste streams into high-value lipids not only reduces production costs but also embodies the principles of the circular bioeconomy.

Sustainable industrialization requires a holistic optimization of the downstream cascade, including biomass harvesting, cell wall disruption, and lipid extraction. As discussed in Sect.  6, the adoption of green, energy-efficient separation technologies is essential to minimize product loss and environmental footprint. Additionally, the application of TEA methods can be instrumental in assessing the production costs of GLA-producing strains. This analytical approach facilitates the optimization of production workflows and cost-reduction strategies, thereby paving the way for the industrial-scale production of GLA. In our recent investigation (Yang et al. 2025), we conducted a comprehensive TEA to evaluate the commercial viability of microbial GLA produced from corn stover. Based on a Gaussian Process-driven process simulation, we estimated a competitive minimum selling price of $17,821 per ton for GLA oil. Furthermore, our sensitivity analysis identified utility costs, feedstock prices, and enzyme costs as the primary economic drivers, offering precise targets for future cost-reduction efforts. Ultimately, such data-driven economic assessments can guide future optimization by focusing efforts on the most cost-sensitive bottlenecks, thereby accelerating the commercialization of microbially sourced GLA.

Overall, the future development of microbial GLA production will require the coordinated integration of advanced strain engineering, fermentation optimization, sustainable feedstocks, efficient downstream processing, and techno-economic assessment. Such an integrated approach will be essential for translating improvements in GLA biosynthesis into cost-effective and scalable industrial production.

## Data Availability

There are no new data associated with this article.
